# Editorial: The interface between social psychology and educational psychology: interactional phenomena in educational settings

**DOI:** 10.3389/fpsyg.2026.1812864

**Published:** 2026-03-09

**Authors:** Eva Hammar Chiriac, Gisela Steins, Tilmann Wilton

**Affiliations:** 1 Department of Behavioural Sciences and Learning, Linkoping University, Linköping, Sweden; 2 Faculty of Educational Sciences, Universitat Duisburg-Essen, Essen, Germany

**Keywords:** educational psychology, educational context, pedagogical field, interpersonal interaction, group dynamics, social psychology, social psychology theories

Applying social psychological theories to the dynamics and challenges of teaching and learning environments has a long-standing tradition. As early as 1933, systematic insights on this subject were compiled by Feldman (1990). Overall, this tradition has been persistently followed (e.g., Babad, 2009), but it is a path that can easily be overlooked. With this research focus, we aim to help keep this path alive and visible. Since we believe that social psychological perspectives can be highly beneficial when applied to the pedagogical field, this Research Topic aims to bridge the gap between social psychology and educational psychology in educational settings. Given the crucial role of interactions and dynamics in educational environments, it is valuable to consider the social context, its dynamics, and processes through a social psychological perspective. We are therefore very pleased to have contributed to this field.

## Characteristics of the contributions in the Research Topic

In this Research Topic, we are delighted to present 12 interesting contributions displaying a diverse variation on how social psychological theories can contribute with new perspectives and expand knowledge on pedagogical issues. The edition contains 10 original contributions, one conceptual contribution, and one brief research report. The contributions are characterized by enormous diversity in terms of origin, theoretical and methodological approach, and research questions addressed. While early considerations of applying social psychological theory to questions of teaching and learning and interaction in educational contexts mostly involved systematic applications of theory—a classic example being the many systematic applications of attribution theory (Weiner, 2006)—the interconnections between social psychology and educational psychology in the present contributions are multifaceted and complex. This may be a sign that social psychological concepts have disseminated into Educational Psychology.

The studies in the twelve articles included were conducted (a) in different countries: China, Germany Sweden, Switzerland, Türkiye and the USA, (b) at various levels of the educational context: universities, schools and preschools (c) using a variety of social psychology theories, (d) methods: qualitative (focus group interviews, interviews, observation) and quantitative (questionnaires, path analyses) and mixed-method, and (d) highlight the perspectives of different stakeholders: students, teachers and parents. As the regional contexts, as well as the organization of the contributions, are heterogeneous, many of the findings cannot be transferred. The contributions are compiled under three themes.

## The dynamics between individuals and groups within educational settings

The first theme, which deals with dynamic processes between different stakeholders in the school environment, is also the theme that generated the most contributions in this topic. Two contributions highlight the relevance of parents for their children's success at school. The first contribution by Chwastek et al. provides insights into the importance of increasing Arabic-speaking refugee parents' access to resources to facilitate their academic engagement in elementary schools in lower-income neighborhoods of a higher-income country. Based on middle school students in China, the study of Zhang et al. explores the relationship between parental perceived teacher care and teacher-student relationship and the role of teacher gender. The reader is provided with inspiration on how educators could strengthen the communication and cooperation between parents and teachers.

Additionally, two articles focus on the teacher's perspective. Karakus examines teachers' attitudes and motivations toward distance learning among volunteer pre-service teachers enrolled in a university in Türkiye. This research is relevant for all lecturers involved in distance teacher training.

This theme also includes a contribution from Schroeder et al. (USA) concerning the accuracy of personal perception: How do teachers know the right thing to do next in the classroom? This single conceptual contribution can be used to reflect on one's own knowledge of the accuracy of one's perceptions in relation to perspective taking, regardless of institutional context.

Further, this theme encompasses articles that address various issues within the university teaching and learning setting. Han et al. investigate how Chinese university students perceive classroom justice in relation to students' intention to cyberloaf and elaborate why classroom justice represents a highly cost-effective strategy for reducing student cyberloafing, of great interest to university lecturers. Li investigated the intricate relationships between perceived school climate, challenging job demands, emotion regulation, and teacher burnout among Chinese university instructors for English as a foreign language. This article demonstrates the relevance of such concepts for understanding dynamics of the university teaching and learning setting. Yan et al. examine a well-known group dynamic phenomenon, free-riding, which is closely related to the experience of injustice within the group where it occurs (Chinese students). The authors present practical conclusions about how the resulting and perceived unfairness can be addressed.

## Interaction strategies between educators and students

In the second theme, we have included articles that focus on interpersonal relationship between various stakeholders in the educational setting. The contribution by Feng and Sun deals with the strategic decision-making mechanisms among students, universities, and competition organizers, considered as three players within a tripartite evolutionary game model. The model examines students' innovation decisions shaped by contextual and institutional influences. The readers are provided with insights into how a campus culture can potentially motivate innovation more strongly.

Based on the interpersonal relationship model, the study by Li et al. examines Hong Kong PhD students' perceptions of eight established supervisor–student relationship styles and their impact on students. The findings show that the PhD student-supervisor relationship may have a boosting or hindrance effect for the student. The authors present practical implications for improving PhD student–supervisor relationships.

## Educational processes based on social psychological concepts or backgrounds

The common denominator for the contributions in the third theme is the application of the social psychology framework and/or concepts to offer new insights. Löfstrand and Zakrisson highlight the valuable effects of simulation and role-playing exercises can have on students' cognitive and emotional responses to complex social issues such as stereotyping, prejudice, and asylum seeking. The article is motivating for lecturers hesitant about role-playing as a teaching method. The long-term study by Egger et al. identifies the potentials and limitations of argument-based intervention through narrative fiction regarding social reasoning about immigrant-based exclusion. The findings are exciting for all teaching scenarios in which there is a lot of argumentation and discussion. Finally, Forsberg et al. studied students' perspectives of school climate, student-teacher relationship and gender in the classroom. The results reveal that teachers' school climate work based on taken-for-granted gender norms reinforces unfair, differentiated, fragmented classroom management practices.

## Conclusion

Together, the collection of articles in this Research Topic contributes new knowledge and a deeper understanding of the research field, but also offers new insights for experienced practitioners through its social-psychological framework. In the hope that these findings will have an impact on more research and practice at the interface between social psychology and educational psychology and thereby keep this path alive and visible, we wish our readers an enjoyable reading!
